# Impact of Infection-Related Immunosuppressant Reduction on Kidney Transplant Outcomes: A Retrospective Study Considering the Temporal Dynamics of Immunosuppressive Requirements

**DOI:** 10.3389/ti.2023.11802

**Published:** 2023-11-21

**Authors:** Bo Yang, Qianqian Ye, Changhao Huang, Xiang Ding

**Affiliations:** ^1^ Department of Organ Transplantation, Xiangya Hospital, Central South University, Changsha, Hunan, China; ^2^ Department of Pharmacy, Xiangya Hospital, Central South University, Changsha, Hunan, China; ^3^ Liver Cancer Laboratory, Xiangya Hospital, Central South University, Changsha, Hunan, China

**Keywords:** kidney transplantation, graft survival, infection, immunosuppressant reduction, rejection

## Abstract

Immunosuppressant reduction (ISR) is a common treatment for kidney transplant recipients experiencing infections, but its impacts on kidney transplant outcomes remains unclear. This retrospective single-center study included 300 patients who underwent kidney transplantation between January 2017 and April 2020. The post-transplant timeline was divided into four distinct phases: ≤1 month, 2–6 months, 7–12 months, and >12 months. Patients were categorized based on the presence of clinically relevant infections and whether they received ISR. Significant differences were observed in the spectrum of clinically relevant infections across the post-transplant phases. During the ≤1 month phase, primary infections were associated surgical operation, such as urinary tract infections involving *Enterococcus* spp. and *Candida* spp. Cytomegalovirus and BK polyomavirus (BKPyV) infections increased during the 2–6 months and 7–12 months periods. Approximately one-third of patients experienced ISR due to infection, with BKPyV infections being the primary causes. Recipients who experienced their first ISR due to infection between 2–6 months and 7–12 months had worse graft survival comparing with patients without any infections. ISR due to infections between 2 and 6 months was associated with a higher risk of rejection. Tailored ISR strategies should be developed according to temporal dynamics of immunosuppressive intensity to prevent rejection.

## Graphical Abstract

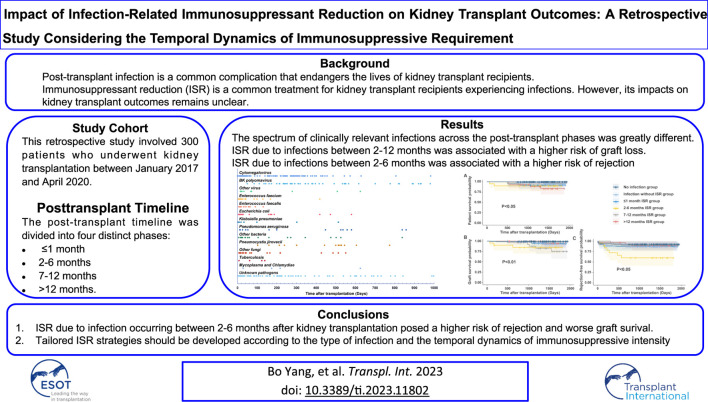

## Introduction

Post-transplant infection is a common complication that endangers the lives of kidney transplant recipients. Numerous studies have reported that up to 80% of patients experience at least one episode of infection during the first year following transplantation [1, 2]. Despite the administration of post-operative prophylaxis, infections still account for approximately 21% of deaths during long-term follow-up and remain the most common non-cardiovascular cause of death after kidney transplantation [3, 4].

Successful treatment of post-transplant infections requires accurate diagnosis, targeted antimicrobial therapy, and effective critical care support. Additionally, reducing the intensity of immunosuppression through immunosuppressant reduction (ISR) has been proposed to improve patient’s recovery from post-transplant infections [5, 6]. Nevertheless, this strategy poses the risk of acute rejection, as the appropriate extent and duration of ISR are difficult to determine. Previous studies examining the relationship between ISR due to infection and the risk of rejection have yielded conflicting results. While some studies suggest that ISR does not increase the risk of graft loss or acute rejection in patients with bacterial infections, severe pneumonia, or BK polyomavirus (BKPyV) infection [7–10], others suggest that kidney recipients with ISR due to infection may be more susceptible to rejection [11, 12]. Moreover, the impacts of clinical factors such as the time, duration, and methods of ISR on the risk of rejection are not fully understood.

It is generally acknowledged that higher concentrations of immunosuppressants are required in the early phase after transplantation to prevent rejection. Earlier studies that aimed at reducing the toxicity of immunosuppressants indicated that early reductions in tacrolimus (TAC) or mycophenolate mofetil (MMF) levels after kidney transplantation were linked to a higher rejection risk [13–15]. Conversely, late-phase reductions in TAC or MMF were relatively safer [16, 17]. Based on these findings, we hypothesize that patients who receive ISR due to infection early after kidney transplantation may be at a higher risk of rejection. To explore this hypothesis, we conducted a retrospective study on a cohort of consecutive patients who underwent deceased donor kidney transplantation in our center. The objective of this study is to identify the association between ISR due to infection at different phases after transplantation and the risk of rejection.

## Materials and Methods

### Study Design

The organ donation and procurement protocols were approved by the ethics committee of Xiangya Hospital, Central South University. Written, informed consent was obtained from all donors and recipients. No executed prisoners’ donations were used following international human rights guidelines of the Declaration of Helsinki and the Declaration of Istanbul.

This study enrolled a total of 300 consecutive patients who underwent deceased donor kidney transplantation at our center between January 2017 and April 2020. The perioperative clinical and laboratory data of both donors and recipients were obtained from their medical records and the Chinese Scientific Registry of Kidney Transplantation. Patients with pre-existing donor-specific antibodies (DSA) or those who received multiple organ transplants were excluded. Additionally, we excluded two patients who experienced graft loss shortly after transplantation due to vascular thrombosis, and two patients with primary graft non-function.

According to the protocols of our immunosuppressive therapy and previous studies on the timeline of post-transplant infections [18], we categorized the post-transplant timeline into four phases: ≤1 month, 2–6 months, 7–12 months, and >12 months. Patients were grouped based on the presence of post-transplant infections and whether they received ISR for the infection during different phases (i.e., no infection, infection without ISR, or ISR due to infection within each timeline phase). Patients who had multiple infections with ISR were grouped based on the time of their first infection requiring ISR. Table 1 provides an overview of the baseline and post-transplant clinical characteristics of patients.

**TABLE 1:** The baseline characteristics and post-transplant complications of different groups.

Patients’ characteristics	No infection (group 1, *n* = 129)	Infection (*n* = 171)						*p*-value
		Without ISR (group 2, *n* = 68)	Time phases of first ISR due to infection (*n* = 103)					
			≤1 month(group 3, *n* = 25)	2–6 months(group 4, *n* = 20)	7–12 months (group 5, *n* = 30)		>12 months(group 6, *n* = 28)	
Age (years), mean ± SD	42.3 ± 10.8	38.8 ± 10.2	45.0 ± 11.9	41.3 ± 13.3		40.3 ± 10.5	42.9 ± 12.1	0.152
Male sex, *n* (%)	100 (77.5)	47 (69.1)	16 (64.0)	17 (85.0)		13 (43.3)^a^ ^,^ ^b^ ^,^ ^c^	19 (67.9)	<0.01
PRA positive, *n* (%)	3 (2.3)	3 (4.4)	0 (0.0)	2 (10.0)		2 (6.7)	1 (3.6)	0.317
HLA mismatches, mean ± SD	3.8 ± 1.3	3.6 ± 1.4	3.6 ± 1.2	3.3 ± 1.2		3.8 ± 0.9	3.7 ± 1.3	0.517
First Transplantation, *n* (%)	120 (93.0)	65 (95.6)	23 (92.0)	19 (95.0)		29 (96.7)	27 (96.4)	0.957
Leading causes of ESRD, *n* (%)								
Chronic nephritis^d^	85 (65.9)	57 (83.8)	18 (72.0)	17 (85.0)		24 (80.0)	18 (64.3)	0.057
Diabetic nephropathy	11 (8.5)	2 (2.9)	2 (8.0)	0 (0.0)		1 (3.3)	1 (3.6)	0.545
IgA nephropathy	9 (7.0)	2 (2.9)	1 (4.0)	0 (0.0)		2 (6.7)	4 (14.3)	0.326
Hypertensive nephrosclerosis	8 (6.2)	1 (1.5)	2 (8.0)	1 (5.0)		2 (6.7)	0 (0.0)	0.364
Polycystic kidney	5 (3.9)	2 (2.9)	1 (4.0)	1 (5.0)		0 (0.0)	2 (7.1)	0.717
History of blood transfusion, *n* (%)	7 (20.9)	13 (19.1)	5 (20.0)	4 (20.0)		6 (20.0)	7 (25.0)	0.993
History of smoking	18 (14.0)	19 (27.9)	5 (20.0)	6 (30.0)		3 (10.0)	6 (21.4)	0.132
Pre-transplant comorbidities, *n* (%)								
Essential hypertension	37 (28.7)	13 (19.1)	10 (40.0)	6 (30.0)		8 (26.7)	7 (25.0)	0.469
Type 2 diabetes	17 (13.2)	4 (5.9)	5 (20.0)	2 (10.0)		1 (3.3)	1 (3.6)	0.156
Coronary heart disease	5 (3.9)	3 (4.4)	3 (12.0)	1 (5.0)		1 (3.3)	4 (14.3)	0.185
Hepatitis B viral infection	16 (12.4)	7 (10.3)	4 (16.0)	0 (0.0)		4 (13.3)	3 (10.7)	0.595
History of tuberculosis	2 (1.6)	1 (1.5)	2 (8.0)	1 (5.0)		1 (3.3)	1 (3.6)	0.250
Hemoglobin (g/L), mean ± SD	111.7 ± 21.8	109.8 ± 20.7	107.2 ± 22.4	104.5 ± 14.7		104.7 ± 15.5	108.2 ± 22.3	0.452
Cold ischemia time (h), mean ± SD	10.4 ± 3.5	10.5 ± 3.4	11.1 ± 2.9	11.3 ± 4.6		10.1 ± 4.1	10.4 ± 3.6	0.835
Induction therapy, *n* (%)								
Basiliximab	73 (56.6)	38 (55.9)	15 (60.0)	8 (40.0)		19 (63.3)	17 (60.7)	0.687
ATG	51 (39.5)	25 (36.8)	9 (36.0)	11 (55.0)		11 (36.7)	10 (35.7)	0.766
Baciliximab + ATG	5 (3.9)	5 (7.4)	1 (4.0)	1 (5.0)		0 (0.0)	1 (3.6)	0.707
Post-transplant complications, *n* (%)								
DGF	18 (14.0)	13 (19.1)	6 (24.0)	8 (40.0)		6 (20.0)	3 (10.7)	0.101
Urinary fistula	0 (0.0)	8 (11.8)^a^	3 (12.0)^a^	4 (20.0)^a^		1 (3.3)	0 (0.0)^c^	<0.01
Ureteral stenosis	0 (0.0)	1 (1.5)	0 (0.0)	1 (5.0)		0 (0.0)	1 (3.6)	0.081
NODAT	5 (3.9)	6 (8.8)	5 (20.0)	2 (10.0)		3 (10.0)	4 (14.3)	0.051
All Rejections (*n*, %)	10 (7.8)	9 (13.2)	3 (12.0)	7 (35.0)^a^ ^,^ ^b^		3 (10.0)	3 (10.7)	0.049
Post-infection rejection	—	6 (8.8)	3 (12.0)	7 (35.0)^b^		3 (10.0)	2 (7.1)^c^	0.038
TCMR	8 (5.6)	2 (2.9)	2 (8.0)	2 (10.0)		1 (3.7)	0 (0.0)	0.624
ABMR	2 (1.4)	3 (4.4)	0 (0.0)	3 (15.0)^a^		1 (3.7)	2 (11.1)	0.020
Mixed rejection	0 (0.0)	1 (1.5)	1 (4.0)	2 (10.0)^a^		1 (3.7)	0 (0.0)	0.015

Abbreviations: ISR, immunosuppressants reduction; CMV, cytomegalovirus; PRA, panel reactive antibodies; HLA, human leukocyte antigen; ESRD, end stage renal disease; ATG, antithyroglobulin; DGF, delayed graft function; NODAT, new onset diabetes after transplantation; TCMR, T cell-mediated rejection; ABMR, antibody-mediated rejection.

^a^ Significant different from group 1.

^b^ Significant different from group 2.

^c^ Significant different from group 4.

^d^ Diagnosed based on clinical manifestation and laboratory findings, without renal biopsy.

### The Protocols of Immunosuppressive Induction and Maintenance Therapy

Anti-thymocyte globulin (ATG) induction therapy involved administering 50 mg/day of ATG at the time of transplantation and for the next 2 days; alternatively, two doses of 20 mg basiliximab were injected during the operation and on the fourth day post-transplant. In some patients with delayed graft function (DGF), basiliximab was initially given upon transplantation but then switched to ATG within the following 3 days to mitigate ischemia-reperfusion injury. Maintenance immunosuppressive therapy consisted of a TAC-based regimen in combination with prednisone and MMF. The TAC target concentration was 10–12 ng/mL within the first month, 8–10 ng/mL between the 2nd and 6th month, 6–8 ng/mL from the 7th to the 12th month, and above 5 ng/mL thereafter. The initial dose of MMF was 1.5 g/day and tapered to 1 g/day after 1 year. Methylprednisolone (500 mg/day) was administered on the day of the procedure and for the next 2 days, followed by oral prednisone starting at 40 mg/day and gradually tapered to 10 mg/day at 1 month and 5 mg/day at 1 year.

### The Prophylaxis Protocols for Post-Transplant Infections

Cefoperazone-sulbactam or piperacillin-tazobactam was routinely administered at the time of transplantation and was discontinued within 5 days if there was no sign of infection. Trimethoprim/Sulfamethoxazole (SMZ) was used for prophylaxis (0.48 g/day) against *Pneumocystis jiroveci* pneumonia (PJP) for 6–12 months after transplantation. Since valganciclovir was not covered by most health insurance in China, ganciclovir (1.5 g/day) was given to most patients for 3–6 months to prevent Cytomegalovirus (CMV) infection. For patients who had DGF or could not tolerate ganciclovir, CMV viremia was monitored monthly for 6 months, and then every 3–6 months thereafter. Pre-emptive treatment was initiated when CMV viremia was detected. Among patients with rejection, SMZ and ganciclovir were used for 3 months to prevent infections after antirejection treatments.

A Double J stent was routinely inserted during transplantation to prevent urinary complications and was typically removed after 3–4 weeks, with the aim of reducing the risk of urinary tract infections (UTI). In cases involving patients with a urinary fistula, the removal of the stent was deferred until the leakage had healed.

### The Diagnosis of Post-Transplant Clinically Relevant Infections and Rejection

The clinically relevant infection was defined as previously described with minor modification [19]. Briefly, bacterial infections require the isolation of a bacterial pathogen, clinical signs/symptoms, and specific antibiotic treatment. Clinically relevant fungal infections require histopathology of a tissue biopsy showing invading fungal hyphae or yeasts, or clinical and microbiological criteria (probable invasive fungal infection by European Organization for Research and Treatment of Cancer definition) [20, 21]. CMV infection was considered clinically relevant when viremia or CMV disease was evidenced [22]. BKPyV infection was classified as clinically relevant if it was confirmed through biopsy as BKPyV nephropathy or presumed BKPyV nephropathy based on viral load criteria [23]. Alternatively, patients with BKPyV infection were subjected to pre-emptive ISR treatment [9]. Other clinically relevant viral infections are defined by the detection of viral replication together with clinical signs/symptoms. Infections with unknown pathogens were considered clinically relevant when they were symptomatic, evidenced by imaging and/or other laboratory examinations, and required antibiotic treatment.

The indication biopsy was performed on patients with allograft dysfunction, and the pathological diagnosis of rejection and BKPyV nephropathy was based on Banff criteria.

### The Strategy of Reduction and Resumption of Immunosuppressants During the Treatment of Infection

The ISR due to infection was defined as any immunosuppression reduction as a part of treatment for any post-transplant infection. The criteria for applying ISR in patients with pulmonary infection is based on the Infectious Diseases Society of America/American Thoracic Society (IDSA/ATS) 2007 guidelines for severe pneumonia [24]. In cases where patients do not meet IDSA/ATS criteria, ISR may be applied if potential life-threatening infections are suspected, or if there is no improvement in key symptoms and indicators, including fever, dyspnea, and PaO2/FIO2 ratio, after 48 h of antibiotic treatment. The approach to reinstating immunosuppressants was tailored to the type of pathogen and individual patient’s condition. Standard prerequisites for reintroducing immunosuppressants include an absence of fever for at least 72 h, significant improvements in pulmonary function, and improved chest X-ray results. The initial step typically involves gradually resuming the calcineurin inhibitor (CNI) to its prescribed concentration. Once a sustained decrease or cessation of infection is confirmed, MMF may be reintroduced progressively at its customary dose.

For patients with simple CMV viremia or UTI, reductions in immunosuppressants were considered if over-immunosuppression was suspected based on clinical experience and laboratory test results. Tapering of MMF was typically the first step, and it was resumed when the infection had been cured for at least 1 week. For patients with complicated conditions such as repeated infections due to multi-drug-resistant strains, mixed fungal and bacterial infections, etc., the strategy of ISR was determined by physicians according to their clinical judgment of the patient’s condition.

BKPyV replication was monitored monthly for 6 months after transplantation, followed by subsequent monitoring every 3–6 months in the absence of BKPyV infection. Detection of active viral infection, defined as a viral load of >2 × 10^3^ copies/mL in urine, prompted a thorough review of the patient’s immunosuppressive regimen, and appropriate adjustments were made according to our center’s established practices. Moreover, urine and blood BKPyV detection was repeated every 3 weeks to monitor trends and inform further decisions. If urine BKPyV replication increased rapidly, or blood BKPyV viremia was detected, ISR was applied to the affected patients as needed. ISR involved halving the dosage of MMF and decreasing CNI levels to 4–5 ng/mL for TAC or 80–100 ng/mL for cyclosporine (CsA). For patients with declining graft function due to BKPyV nephropathy or no improvement in BKPyV replication following CNI and MMF reduction, MMF was replaced by Leflunomide at a dose of 20 mg/day. Additionally, intravenous immunoglobulins were administered monthly at a dosage of 0.1–0.2 g/kg for at least 4 months. After BKPyV infection, we considered increasing the intensity of immunosuppression among patients with stable graft function. However, this was only done when their viral load was undetectable in blood and <1 × 10^4^ copies/mL in urine for at least two consecutive months. In such cases, the CNI level could be increased to 5–6 ng/mL for TAC or 100–120 ng/mL for CsA. If the graft function remained stable and the viral load continued to improve, MMF could replace Leflunomide.

### Follow-Up

All patients regularly visit our clinics as required for monitoring graft function and drug concentration. Whenever major complications were suspected, the patients were admitted to the hospital for further examination. To minimize the impact of COVID-19 on our study, the follow-up date was set to 30 April 2022.

### Statistical Analysis

Continuous variables were analyzed by Analysis of Variance and summarized as mean ± standard deviation (SD). Categorical variables were analyzed by chi-square or Fisher’s exact tests with Bonferroni correction and described using frequencies and percentiles. Accumulate survival rates were calculated by the life table. Survival curves were estimated by the Kaplan–Meier method and compared with the log-rank tests. Analysis of risk factors was determined by binary logistic regression. SPSS software 23.0 (IBM, United States) was used for data analysis, and *p*-value < 0.05 was considered to be statistically significant. All tests were 2-tailed.

## Results

### The Baseline Demographic and Clinical Characteristics of Patients in Different Groups

A total of 171 patients (57.0%) experienced 413 clinically relevant infections during the follow-up period of this study. Among the infection group, 103 out of 171 patients (60.2%) received ISR due to infection. Table 1 showed that the baseline characteristics of the groups were largely similar, except for a lower proportion of male patients in the 7–12 months ISR group (43.3%) compared to the no infection group (77.5%, *p* < 0.05), infection without ISR group (69.1%, *p* < 0.05), and the 2–6 months ISR group (85.0%, *p* < 0.05). Regarding post-transplant complications, the incidence of urinary fistula was significantly lower (*p* < 0.05) in the no infection group (0.0%, 0/129) compared to the infection without ISR group (11.8%, 8/68), the ≤1 month ISR group (12.0%, 3/25), and the 2–6 months ISR group (20.0%, 4/20). Furthermore, the incidence of urinary fistula in the >12 months ISR group (0.0%, 0/28) was significantly lower than that in the 2–6 months ISR group (*p* < 0.05). The incidence of rejection was significantly higher (*p* < 0.05) in the 2–6 months ISR group (35%, 7/20) compared to the no infection group (7.8%, 10/129) and the infection without ISR group (13.2%, 9/68). Majority of these rejections (60.0%, 21/35) occurred after post-transplant infections.

### The Characteristics of Post-Transplant Infections at Different Phases After Transplantation

**FIGURE 1:**
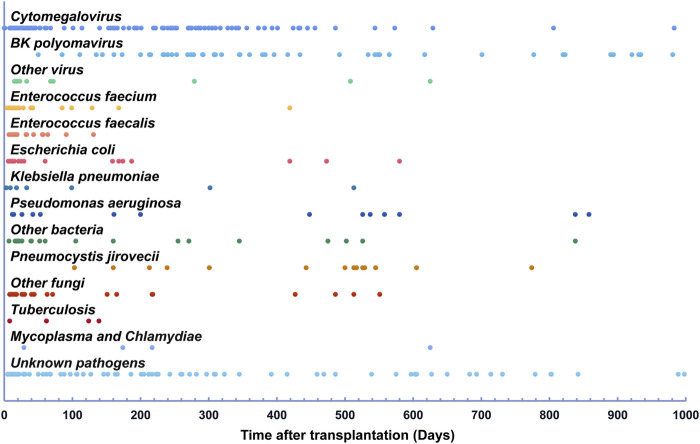
Infections detected within 1,000 days after renal transplantation.

Figure 1 and Table 2 present a detailed timeline and characteristics of infections following kidney transplantation. Within the first month post-transplantation, there were 106 clinically relevant infections in 80 patients, with UTIs accounting for 49.1% of all infections, significantly higher than other time phases (*p* < 0.05). However, no BKPyV infections were identified during this phase. Between 2 and 6 months post-transplantation, 62 patients experienced 95 clinically relevant infections, with the proportion of UTIs to all infections decreasing to 29.5% compared to the first month (49.1%, *p* < 0.05), while the incidence of clinically relevant BKPyV infection increased to 7.4%. During the period between 7 and 12 months post-transplantation, 67 patients experienced 88 clinically relevant infections, with BKPyV infection constituting 25.0% of all infections, which was much higher than the first month (0.0%, *p* < 0.01) and 2–6 months post-transplantation (7.4%, *p* < 0.05). The proportion of UTI was lowest in the 7–12 months phase (9.1%), and this difference was statistically significant when compared to previous time phases (*p* < 0.05). From the 13th month to the end of the follow-up, 82 patients experienced 124 clinically relevant infections. Pulmonary infection (25.2%) and BKPyV infection (28.5%) were the two most common infections during this period.

**TABLE 2:** The types of infections in different time phases after transplantation.

Types of infection	Infection cases at different post-transplant time phases				*p*-value
	≤1 month (Group 1, *n* = 106)	2–6 months (Group 2, *n* = 95)	7–12 months (Group 3, *n* = 88)	>12 months (Group 4, *n* = 124)	
Pulmonary infections, *n* (%)	21 (19.8)	25 (26.3)	22 (25.0)	31 (25.2)	0.697
Urinary tract infections, *n* (%)	52 (49.1)	28 (29.5)^a^	8 (9.1)^a^ ^,^ ^b^	20 (16.3)^a^ ^,^ ^b^	<0.001
BKPyV infection, *n* (%)	0 (0.0)	7 (7.4)^a^	22 (25.0)^a^ ^,^ ^b^	35 (28.5)^a^ ^,^ ^b^	<0.001
CMV viremia, *n* (%)	19 (17.9)	29 (30.5)^a^	33 (37.5)^a^	21 (17.1)^b^ ^,^ ^c^	0.001
Other sites, *n* (%)	10 (9.4)	5 (5.3)	3 (3.4)	16 (13.0)	0.050
Surgical wound, *n* (%)	4 (3.8)	1 (1.1)	0 (0.0)	0 (0.0)^a^	0.032

Abbreviations: BKPyV, BK polyomavirus; CMV, cytomegalovirus.

^a^ Significantly different from Group 1.

^b^Significantly different from Group 2.

^c^ Significantly different from Group 3.

The proportion of CMV infection among all infections was highest in the 7–12 months phase (37.5%) and lowest in the >12 months phase (17.1%), and this difference was statistically significant (*p* < 0.05). The proportion of pulmonary infections among all infections ranged from 19.8% to 26.3%, and the difference was not statistically significant (*p* > 0.05).

The pathogens identified in post-transplant infections were summarized in Supplementary Table S1. Regarding gram-positive bacterial pathogens, *Enterococcus faecalis* and *Enterococcus faecium* were frequently identified, accounting for 43.4% and 46.9% of all bacterial pathogens isolated during the ≤1 month and 2–6 months post-transplant phases, respectively. These *Enterococcus spp*. were mainly implicated in UTIs within the first 3 months after transplantation but were rare thereafter. The commonly isolated gram-negative bacterial pathogens following kidney transplantation were *Pseudomonas aeruginosa*, *Klebsiella pneumoniae*, and *Escherichia coli*. *Pseudomonas aeruginosa* was mainly found in patients with pulmonary infections and accounted for 6.3%–16.7% of all isolated bacterial pathogens in each post-transplant phase, respectively. *Klebsiella pneumoniae* was a substantial pathogen for both pulmonary infections and UTIs, accounting for 7.5%–16.7% of all isolated bacterial pathogens in ≤1 month, 2–6 months, and 7–12 months post-transplant phases, but comprised 45% of all bacterial pathogens isolated in the >12 months post-transplant phase. In contrast, *Escherichia coli* emerged as a major pathogen for UTIs, responsible for 22.6% of all bacterial pathogens isolated within the first month after transplantation, significantly higher than *Pseudomonas aeruginosa* (7.5%) and *Klebsiella pneumoniae* (7.5%). *Candida spp*. were primarily found in the early post-transplant phases, representing 87.5% of fungal infections within the first month after transplantation, but became less prevalent after 3 months. *PJP* reached its peak during 12–24 months after transplantation, accounting for 66.7% of all fungal infections between 13 months to the end of follow-up and was mostly following cessation of SMZ.

### The Characteristics of ISR Due to Infections at Different Phases After Transplantation

Among the 103 patients with infection-triggered ISR, 29 had experienced two episodes of ISR, and 3 patients had three episodes (Table 3). The initial episode of ISR was attributed to pulmonary infections in 47.6% (49/103) of cases, while BKPyV infections and UTIs accounted for 25.2% (26/103) and 19.4% (20/103) of cases, respectively. Only one patient experienced initial ISR as a result of CMV infection. In contrast, BKPyV infection was the predominant cause (60.0%, 21/35) of repeated ISR (detailed in Table 3 and Supplementary Table S2) while UTIs were responsible for only two cases of repeated ISR.

**TABLE 3:** The characteristics of ISR due to infection in different time phases after transplantation.

Characteristics	Patients with ISR due to infection (*n* = 103)				*p*-value
	≤1 month (group 1, *n* = 25)	2–6 months (group 2, *n* = 20)	7–12 months (group 3, *n* = 30)	>12 months (group 4, *n* = 28)	
Infections leading to first ISR, *n* (%)					
Pulmonary infection	10 (40.0)	12 (60.0)	16 (53.3)	11 (39.3)	0.410
Urinary tract infection	12 (48.0)	5 (25.0)	2 (6.7)^a^	1 (3.6)^a^	<0.001
BKPyV infection	0 (0.0)	2 (10.0)	11 (36.7)^a^ ^,^ ^b^	13 (46.4)^a^ ^,^ ^b^	<0.001
Other infections	3 (12.0)	1 (5.0)	1 (3.3)	3 (10.7)	0.590
Patients with ISW, *n* (%)	3 (12.0)	5 (25.0)	6 (20.0)	8 (28.6)	0.498
Patients with repeated ISR, *n* (%)	8 (32.0)	6 (30.0)	12 (40.0)	6 (21.4)	0.507
Infections leading to repeated ISR, *n* (%)					
Pulmonary infection	3 (12.0)	1 (5.0)	4 (13.3)	1 (3.6)	0.534
Urinary infection	1 (4%)	1 (5.0)	0 (0.0)	0 (0.0)	0.343
BKPyV infection	6 (24.0)	4 (20.0)	7 (23.3)	4 (14.3)	0.822
Other infections	0 (0.0)	1 (5.0)	1 (3.3)	1 (3.6)	0.881
Duration of first ISR (days), mean ± SD	35.12 ± 78.1	54.6 ± 141.6	221.9 ± 328.9^a^	278.9 ± 345.7^a^ ^,^ ^b^	0.002
Pulmonary infection	26.3 ± 30.9	30.3 ± 29.1	43.1 ± 70.7	26.27 ± 21.6	0.748
Urinary tract infection	15.6 ± 13.4	9.6 ± 7.0	6.5 ± 3.5	4	0.546
BKPyV infection	—	336.5 ± 439.1	496.7 ± 394.2	560.9 ± 326.8	0.702
Other sites	142.7 ± 218.7	7.0	491.0	74.7 ± 44.1	0.248

Abbreviations: ISR, immunosuppressants reduction; ISW, immunosuppressants withdrawal (completely stopping TAC and MMF); BKPyV, BK polyomavirus.

^a^ Significantly different from group 1.

^b^ Significantly different from group 2.

A total of 22 patients discontinued both CNI and MMF during infection treatment. Among them, one patient halted both CNI and MMF due to sepsis resulting from a UTI, and another patient suspended treatment due to HBV infection, which led to fulminant hepatitis. The remaining 20 patients temporarily suspended CNI and MMF due to severe pulmonary infections. The proportion of patients who temporarily discontinued both medications was 12.0% (3/25), 25.0% (5/20), 20% (6/30), and 28.6% (8/28) in the ≤1 month ISR, 2–6 months ISR, 7–12 months ISR, and >12 months ISR groups, respectively, with no significant differences observed. Additionally, the duration of ISR was significantly shorter in the ≤1 month ISR group and 2–6 months ISR group compared to later time periods. However, the durations of ISR were similar across different groups when categorized by infection type (Table 3). No substantial distinctions in ISR duration were observed between patients with and without rejection (191.3 ± 291.1 vs. 153.8 ± 279.6 days, *p* = 0.63) and between patients with and without graft loss (162.1 ± 284.0 vs. 133.1 ± 252.0 days, *p* = 0.77).

### ISR Due to Infections Was Associated With a Higher Risk of Rejection and Inferior Patient and Graft Survival After Transplantation

**FIGURE 2:**
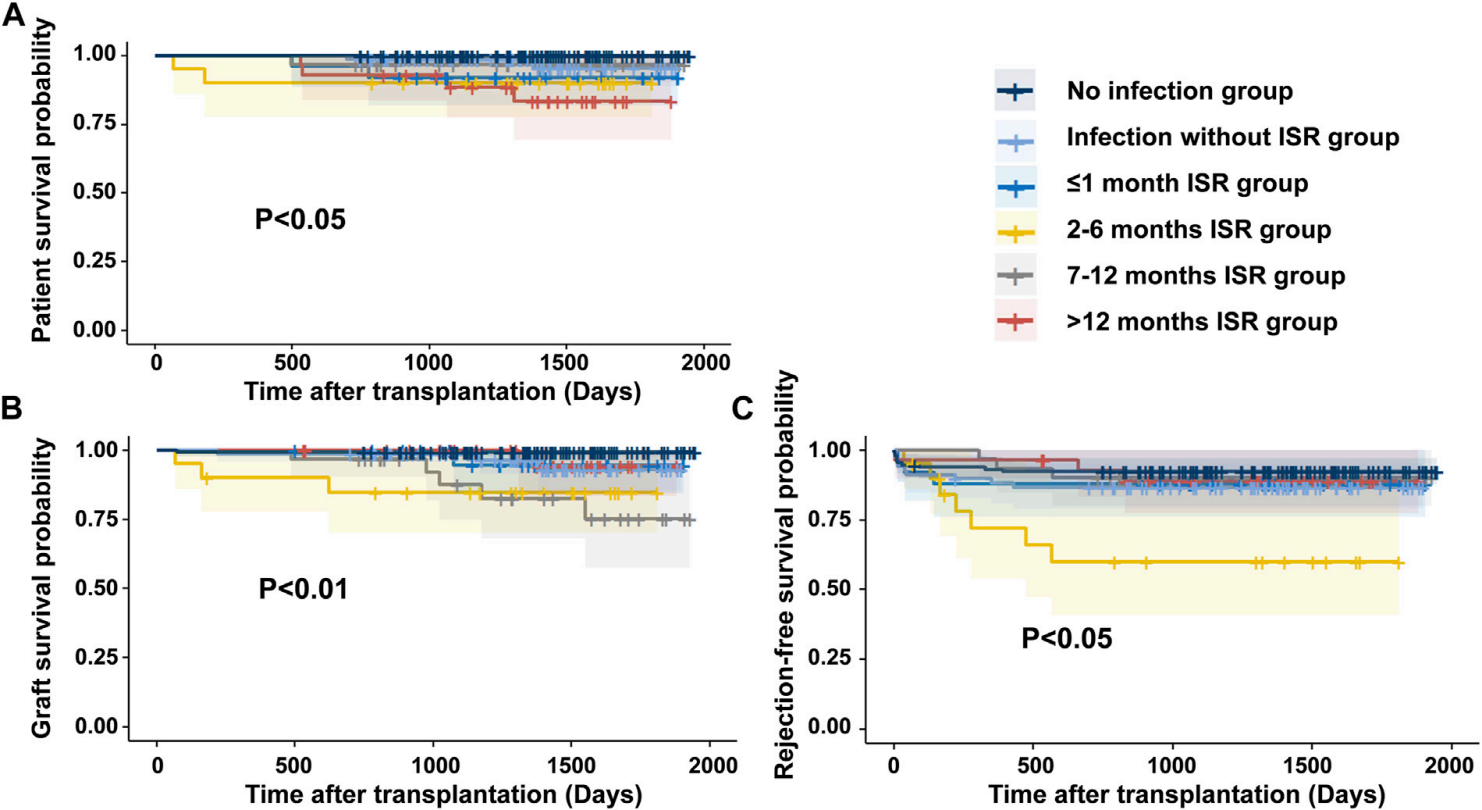
The patient and graft outcomes were associated with ISR due to infection **(A)** The patient survival rate was significantly lower in the >12 months ISR group compared to the no infection group (*p* < 0.01), Infection without ISR group (*p* = 0.049), and the 7–12 months ISR group (*p* = 0.046). **(B)** The death censored allograft survival significantly lower in 2–6 months ISR group and 7–12 months ISR group compared with no infection group. **(C)** Rejection-free survival was inferior in the 2–6 months ISR group compared to all other groups (*p* < 0.05), except for the ≤1 month ISR group (*p* = 0.059).

Figure 2 illustrates the impact of ISR due to infection on patient and graft survival. The 3 years patient survival rates showed a significant difference among the subgroups. Specifically, the no infection group and 7–12 months ISR group had a 100% survival rate, while the infection without ISR group had a 98.5% survival rate, the ≤1 month ISR group had a 92% survival rate, the 2–6 months ISR group had a 90% survival rate, and the >12 months ISR group had an 88.6% survival rate. At 5 years, the survival rates decreased to 83.4% in the >12 months ISR group, while the patients’ survival rates in all other groups remained unchanged. Notably, the no infection group demonstrated significantly higher patient survival rates compared to most subgroups (log-rank *p* < 0.05) except for the 7–12 months ISR group. The 5 years patient survival rate was significantly lower in the >12 months ISR group compared to the no infection group (*p* < 0.01), infection without ISR group (*p* = 0.049), and the 7–12 months ISR group (*p* = 0.046).

Regarding death-censored graft survival rates, at 3 years, the rates were 99.2%, 98.5%, 94.4%, 84.7%, 90.5%, and 100% in the no infection group, infection without ISR group, ≤1 month ISR group, 2–6 months ISR group, 6–12 months ISR group, and the >12 months ISR group, respectively. At 5 years, the graft survival rates decreased to 93.0% in the infection without ISR group, 77.7% in the 6–12 months ISR group, and 94.1% in the >12 months ISR group, but remained unchanged in other groups. The death-censored graft survival rates were significantly lower in the 2–6 months ISR group and 7–12 months ISR group compared to the no infection group (*p* < 0.01), while there was no significant difference between all other subgroups.

During follow-up, the incidence of rejection in the infection group (14.6%, 25/171) was higher than that in the no infection group (7.8%, 10/129; *p* = 0.071). Of the four patients who experienced rejection before infection, three belonged to the infection without ISR group. Among the patients who experienced rejection after infection, 71.4% (15/21) occurred following ISR due to infection. Regarding rejection-free graft survival rates, at 3 years, the rates were 92.2%, 86.7%, 88.0%, 60.1%, 90.0%, and 88.5% in the no infection group, infection without ISR group, ≤1 month ISR group, 2–6 months ISR group, 6–12 months ISR group, and the >12 months ISR group, respectively. At 5 years, rejection-free graft survival rates remained unchanged. The 2–6 months ISR group showed significantly lower rates of rejection-free graft survival compared to all other groups (*p* < 0.05), except for the ≤1 month ISR group (*p* = 0.059).

Multivariate Cox regression analysis identified several factors associated with patient and graft survival. Pulmonary infection (*p* = 0.004), coronary disease (*p* = 0.008), and new onset diabetes after transplantation (*p* = 0.013) were identified as factors associated with patient death (Supplementary Table S3). Moreover, overall infections (*p* = 0.029) were found to be associated with death-censored graft survival (Supplementary Table S4), while PRA positive (*p* = 0.006), ISR due to infections between 2 and 6 months post-transplantation (*p* = 0.035), and smoking history (*p =* 0.012) were identified as factors associated with rejection-free graft survival (Supplementary Table S5).

## Discussion

Currently, there is limited data on the incidence rates of infection-driven ISR. In our study, we observed that 57.0% of patients developed clinically relevant infections, and 34.3% of patients experienced ISR due to infection during follow-up. In Posadas Salas et al.’s study, ISR was defined as sustained TAC levels <8 ng/mL and MMF dosage <1 g/day for at least 1 month within 1 year after transplantation [11]. They reported that 16% of patients had ISR due to infection within the first year after transplantation. As the duration of ISR can vary greatly based on the type, severity, and timing of infections, and physicians often prefer shorter durations of ISR in the early phases after transplantation to minimize the risk of rejection, we did not require a minimum duration of sustained ISR to define ISR, resulting in a higher incidence of ISR due to infection compared to the previous report. Nevertheless, both studies indicate the frequent occurrence of ISR due to infection after transplantation, and further investigation is necessary to understand its impact on patient and graft survival.

To investigate the temporal dynamics of infections and their correlation with rejection, we divided the post-transplant timeline into four phases based on established immunosuppression protocols, antibiotic prophylaxis strategies, and previous research. Our findings revealed significant differences in the spectrum of infections across these phases. In the first month, surgical complications and nosocomial infections were the primary causes of infections, predominantly urinary tract infections involving *Enterococcus* spp. and *Candida* spp. This observation is consistent with prior studies indicating that *Enterococcus* spp. and *Candida* spp. are major infectious pathogens during the early post-transplantation period [2]. Notably, one study found that Beta-lactam antibiotics significantly increase relative gut abundance of *Enterococcus* spp., posing an independent risk factor for *Enterococcal* bacteriuria in kidney recipients [25]. Therefore, our use of Beta-lactam antibiotics for perioperative antibacterial prophylaxis might escalate the likelihood of *Enterococcus* spp. infection. As many of the predisposing factors, such as urinary fistula, prolonged Double J stent placement, and inappropriate antibiotic usage, can be prevented by enhancing surgical techniques and optimizing treatment protocols. Efforts should be made to address these predisposing factors in order to minimize early post-transplant infections and the subsequent need for unnecessary ISR. We observed that CMV and BKPyV infections increased during the 2–6 months and 7–12 months periods, which may be attributed to the maintenance of high immunosuppressive intensity during these periods and cessation of CMV prophylaxis three to 6 months post-transplantation. After 12 months, the incidence of CMV and BKPyV infections declined, likely due to our planned reduction of maintenance immunosuppressive intensity at that point. Similarly, we administered SMZ prophylaxis for 6–12 months, and we noticed an increase in PJP incidence during the 13–24 months post-transplantation following its discontinuation. Our results suggest that the intensity of immunosuppression and antibiotic prophylaxis significantly influence the frequency and timeline of post-transplant infections. As the pharmacokinetic monitoring alone is insufficient for estimating the intensity of immunosuppression after transplantation, researchers have developed some new techniques such as measuring virus-specific T cell levels in addition to pharmacokinetic monitoring, measuring the viral load of torque teno virus, monitoring the intracellular tacrolimus concentration in T-lymphocytes and other immune cells, et al., to solve this problem [26–28]. Optimizing the immunosuppressive protocol based on these new techniques might help us to further reduce the risk of infections and unnecessary ISR in future.

ISR was recommended as the standard treatment for BKPyV infection since no effective antiviral drugs are available [23]. Pre-emptive ISR has demonstrated excellent long-term results for BKPyV infection [9], and our center has implemented a similar strategy. Although some studies have suggested initiating ISR for BKPyV infection upon detection of BKPyV-DNAemia [23], our center aligns with previous findings that indicate sustained BKPyV viruria as an early marker for the development of BKPyV-associated nephropathy. Therefore, our center opts to initiate ISR when the urine BKPyV load is high or shows an increasing trend in subsequent surveillance BKPyV tests [9, 29]. Our data suggest that current ISR strategy for BKPyV infection is effective and does not significantly increase the risk of rejection.

Our results verified the findings of previous research that infection was a major risk for patient and graft survival. Moreover, the survival was worst in patients who had ISR due to infection after 12 months. This could be explained by the fact that more patients had life-threatening pneumonia due to *PJP*, *Klebsiella pneunoniae* infection et al. in the >12 months ISR group. We analyzed the characteristics, including the causes, time, extent, duration, and repeated episodes of ISR due to infection, and investigated their relationship with rejection. We identified that graft survival rates were significantly lower in the 2–6 months ISR group and 7–12 months ISR group compared to the no infection group. ISR during the 2–6 months period due to infection is an independent risk factor for rejection. Our finding did not completely fulfill the previous hypothesis as the earlier time phase (within 1 month after transplantation) was not associated with rejection and a worse prognosis. This could partly be explained by the fact that physicians prefer less extent and shorter durations of ISR in the very early phases after transplantation to minimize the risk of rejection, therefore fewer patients completely stopped TAC and MMF due to infection within 1 month after transplantation. Moreover, the protective effect of induction agents is dose-dependent and weakens over time. Previous studies have shown that induction with low dose ATG (1 mg/kg/day for 3 consecutive days) would result in excellent T cell depletion, but the T lymphocytes will back to normal levels within 1 month after transplantation [30]. As we also use low dose ATG for induction, it is reasonable to think that only patients who had ISR due to infection within 1 month after transplantation might benefit from the rejection-preventing effect of immunosuppressive induction agents, which partly balanced the risk of rejection.

Our study had several limitations. First, due to the retrospective nature of our study, we lacked sufficient data to compare the incidence rates of *de novo* donor specific antibodies (DSAs) between the ISR group and other groups. As DSAs are a major cause of antibody-mediated rejection, we provided detailed pathological diagnostic information in Table 1 to elucidate the link between ISR due to infection and rejection pathology. Additionally, our study cohort was relatively young, potentially limiting its applicability to elderly recipients, who exhibited a lower risk of acute rejection but a higher susceptibility to mortality related to infectious and cardiovascular diseases [31].

In conclusion, our study revealed that ISR due to infection occurring between 2 and 6 months after transplantation may pose a higher risk of rejection, which provides valuable evidence for physicians to adjust their ISR strategy in infection treatment while minimizing the risk of rejection. A tailored ISR strategy should be designed for kidney transplant recipients with post-transplant infections, considering the type of infection and the temporal dynamics of immunosuppressive requirements.

## Data Availability

The data analyzed in this study is subject to the following licenses/restrictions: All the data were supplied by our single-central follow-up study. Requests to access these datasets should be directed to the corresponding author.
