# Supplementary material for: Impact of Infection-Related Immunosuppressant Reduction on Kidney Transplant Outcomes: A Retrospective Study Considering the Temporal Dynamics of Immunosuppressive Requirements
Source: Transpl Int. 2023 Nov 21;36:11802. doi: 10.3389/ti.2023.11802 (PMC10697076; doi:10.3389/ti.2023.11802)
Supplement: Supplementary file 1 [file DataSheet1.docx]

Supplementary table S1. Infectious pathogens identified in different time phases after transplantation.

| Infectious pathogens | Infectious pathogens at different post-transplant time phases | | | | *P*-value |
| --- | --- | --- | --- | --- | --- |
|  | ≤1 month  (group1, n=121) | 2-6 months (group2, n=105) | 7-12 months (group3, n=88) | > 12 months (group4, n=130) |  |
| Viral pathogens, n(%) | | | | | |
| CMV | 21 (17.4) | 30 (28.6) | 33 (37.5) ^a^ | 21 (16.2) ^b,c^ | 0.001 |
| BKPyV | 0 (0.0) | 7 (6.7) ^a^ | 22(23.9) ^a,b^ | 35 (26.9) ^a,b^ | <0.001 |
| Other virus | 4 (3.3) | 3 (2.9) | 1 (1.1) | 5 (3.8) | 0.732 |
| Bacterial pathogens, n(%) | | | | | |
| *Enterococcus faecium* | 15 (12.4) | 7(6.7) | 0 (0.0) ^a,b^ | 1 (0.8) ^a,b^ | <0.001 |
| *Enterococcus faecalis* | 8 (6.6) | 8(7.6) | 0 (0.0) ^a,b^ | 0 (0.0) ^a,b^ | <0.001 |
| *Escherichia coli* | 12 (9.9) | 5(4.8) | 1 (1.1) ^a^ | 3 (2.3) ^a^ | 0.013 |
| *Klebsiella pneunoniae* | 4 (3.3) | 3(2.9) | 1 (1.1) | 9 (6.9) | 0.179 |
| *Pseudomonas aeruginosa* | 4 (3.3) | 2 (1.9) | 1 (1.1) | 2 (1.5) | 0.766 |
| Other bacteria | 10 (8.3) | 7 (6.7) | 3 (3.4) | 5 (3.8) | 0.353 |
| Fungal pathogens, n(%) |  |  |  |  |  |
| *Pneumocystis jiroveci* | 0 (0.0) | 2 (1.9) | 3 (3.4) | 10 (7.7) ^a^ | 0.004 |
| *Candida spp.* | 14 (11.6) | 5(4.8) | 1 (1.1) ^a^ | 0 (0.0) ^a,b^ | <0.001 |
| Other fungus | 2 (1.7) | 3 (2.9) | 1 (1.1) | 5 (3.8) | 0.616 |
| Tuberculosis, n(%) | 1 (0.8) | 3 (2.9) | 0 (0.0) | 1 (0.8) | 0.348 |
| Mycoplasma or Chlamydia, n(%) | 1 (0.8) | 1 (1.0) | 1 (1.1) | 1 (0.8) | 1.000 |
| Unknow, n(%) | 25(20.7) | 19(18.1) | 20(22.7) | 32(24.6) | 0.661 |

a: significant different from group1

b: significant different from group2

c: significant different from group3

Abbreviations: CMV, cytomegalovirus; BKPyV, BK polyomavirus.

Supplementary table S2. the characteristics of ISR due to different types of BKPyV infections

| Patients’ characteristics | BKPyV viruria alone  (N=36) | BKPyV-DNAemia without biopsy  (N=11) | Biopsy proven BKPyV nephropathy | | | *P* |
| --- | --- | --- | --- | --- | --- | --- |
|  |  |  | ClassⅠ Class Ⅱ Class Ⅲ  (N=6) (N=8) (N=3) | | |  |
| Age(years), mean ± SD | 40.5±13.1 | 43.6±12.8 | 47.0±7.4 | 42.0±8.9 | 31.0±5.3 | 0.398 |
| Male sex, n(%） | 18（50.0） | 8（72.7） | 5（83.3） | 5(62.5) | 2(66.7) | 0.499 |
| Patients with ISR, n(%） | 20(55.6) | 11(100.0)^a^ | 5(83.3) | 8(100.0) | 3(100.0) | 0.004 |
| Patients with rejection, n(%） | 5(13.9) | 2(18.2) | 1(16.7) | 1(12.5) | 1(33.3) | 0.826 |
| Duration of ISR (days), mean ± SD | 276.4±181.4 | 874.5±323.8^a^ | 439.4±279.4^b^ | 514.1±299.0^a^ | 415.7±249.3 | 0.000 |

a: significant different from BKPyV viruria alone group

b: significant different from BKPyV-DNAemia without biopsy group

Abrrevations: ISR, immunosuppressants reduction. BKPyV, BK polyomavirus.

Supplementary table S3. Risk factors for patients’ survival

| Risk factors | Univariate | |  | Multivariate | |
| --- | --- | --- | --- | --- | --- |
|  | OR (95%CI) | *P*-value |  | OR (95%CI) | *P*-value |
| Pulmonary infections | 11.907 (2.525-56.141) | 0.002 |  | 9.963 (2.093-47.418) | 0.004 |
| ISR on >12 month | 6.444 (1.818-22.840) | 0.004 |  |  |  |
| Coronary heart disease | 8.390 (2.158-32.613) | 0.002 |  | 5.427 (1.648-26.858) | 0.008 |
| NODAT | 8.137 (2.293-28.881) | 0.001 |  | 5.255 (1.424-19.389) | 0.013 |

Abbreviations: ISR, immunosuppressants reduction; NODAT, new onset diabetes after transplantation.

Supplementary table S4. Risk factors for death-censored graft loss.

| Risk factors | Univariate | |  | Multivariate | |
| --- | --- | --- | --- | --- | --- |
|  | OR (95%CI) | *P*-value |  | OR (95%CI) | *P*-value |
| Infections | 9.755 (1.268-75.041) | 0.029 |  | 9.755 (1.268-75.041) | 0.029 |
| Urinary tract infection | 2.888 (0.970-8.595) | 0.057 |  |  |  |
| ISR due to BKPyV infection | 3.933 (1.078-14.353) | 0.036 |  |  |  |
| ISR with 2-6 month | 4.608 (1.268-16.748) | 0.020 |  |  |  |
| ISR with 7-12 month | 4.191 (1.288-13.635) | 0.017 |  |  |  |
| DGF | 3.039 (0.994-9.293) | 0.051 |  |  |  |

Abbreviations: BKPyV, BK polyomavirus; ISR, immunosuppressants reduction; DGF, delayed graft function.

Supplementary table S5. Risk factors for rejection-free graft survival

| Risk factors | Univariate | |  | Multivariate | |
| --- | --- | --- | --- | --- | --- |
|  | OR (95%CI) | P-value |  | OR (95%CI) | P-value |
| PRA positive | 5.050 (1.955-13.046) | 0.001 |  | 4.052 (1.484-11.067) | 0.006 |
| ISR due to infection between 2-6 months | 4.108 (1.789-9.435) | 0.001 |  | 2.655 (1.069-6.593) | 0.035 |
| Smoking | 2.792 (1.406-5.544) | 0.003 |  | 2.489 (1.223-5.063) | 0.012 |

Abbreviations: PRA, panel reactive antibodies; ISR, immunosuppressants reduction.
